# Hypoglycemic effects of *Acacia nilotica* in type II diabetes: a research proposal

**DOI:** 10.1186/s13104-017-2646-1

**Published:** 2017-07-26

**Authors:** Nasibeh Roozbeh, Leili Darvish, Fatemeh Abdi

**Affiliations:** 1 Mother and Child Welfare Research Center, Hormozgan University of Medical Sciences, Bandar Abbas, Iran; 2 Students Research Committee, Nursing and Midwifery Faculty, Shahid Beheshti University of Medical Sciences, Tehran, Iran

**Keywords:** Hypoglycemia, *Acacia Nilotica*, DiabetesII

## Abstract

**Objective:**

Diabetes mellitus is a common metabolic disorder throughout the world which can negatively affect the function of various body organs. Due to their availability and few side effects, herbal medicines have been proposed as suitable alternatives in the management of diabetes. Previous studies have confirmed the anti diabetic properties of *Acacia nilotica*. The hypoglycemic effects of this plant have been attributed to its role in stimulating the islets of Langerhans to produce more insulin. The present paper describes a systematic review protocol for the assessment of the hypoglycemic effects of *A. nilotica*.

**Main texts:**

Randomized and non-randomized placebo-controlled clinical trials, performed during 1999–2016 will be included. The outcomes will be measured through FBS, GCT, GTT, and OGTT in all of studies and in addition to these tests, will be measured 2HPP and HbA1c level in human study. Well-known databases will be searched for selected key terms *A. nilotica*, type II diabetes and hypoglycemia. The quality assessment of the selected papers will be evaluated based on SYRCLE and Cochrane Risk of Bias Tool. We believe that our findings will provide details about difficulties researchers face during the design of protocols or implementation of scientific studies. Ultimately, the publication of our findings will facilitate the development of effective treatment strategies to promote the health of people with type II DM.

*PROSPERO registration* CRD42016053141

## Introduction

Diabetes mellitus (DM) is one of the most common chronic metabolic disorders throughout the world [1]. Over 180 million are currently affected with type II DM, a disease which is known to double the risk of death in patients [2]. Uncontrolled elevations in blood glucose levels increase oxidative stress which in turn damages cell tissue and causes abnormalities in the structure and function of various body organs [3, 4]. DM interferes with carbohydrate, lipid, and protein metabolism and raises the risk of atherosclerosis by two–six times [1]. Hyperglycemia, a hallmark sign of DM, can be the result of impaired insulin secretion and/or insulin function [5]. A variety of medical and herbal treatments are currently recommended for the management of DM. Despite their efficacy, antidiabetic drugs have raised concerns due to the severe weight loss and increased risk of cardiovascular events following their use [2]. Therefore, owing to their easy availability and fewer side effects, herbal medicines have been suggested as suitable alternatives to antidiabetic drugs [6]. Herbs are known to downregulate blood glucose levels through several pharmacological mechanisms such as lowering carbohydrate absorption, enhancing insulin sensitivity and peripheral glucose uptake, stimulating the secretion of insulin and endogenous incretins, preventing cell apoptosis by exerting antioxidant effects, and promoting glycogenesis or inhibiting glycogenolysis [7, 8]. *Acacia nilotica*, a plant species native to subtropical and tropical areas, is widely found throughout Asia, Africa, and America [9].

It is a branched-chain complex polysaccharide with very low acidity or neutral pH [10]. While *A. nilotica* generally contains (+)-catechin, gallolyated flavan-3, 4-diol, robidandiol, androstene steroid, d-pinitol carbohydrate, catechin-5-galloyl ester, and gallic, m-digallic, and chlorogenic acids, the plants in the Middle East are also rich in potassium, phosphorus, magnesium, iron, and manganese [11]. In the Middle Eastern countries, *A. nilotica* has been traditionally used to treat common cold, cough, fever, gastrointestinal disorders, diarrhea, bloody diarrhea, hemorrhoid, gallbladder problems, ophthalmia, sclerosis, smallpox, tuberculosis, and even cancer. *A. nilotica* wood and leaves have also had applications in the treatment of smallpox and wounds, respectively [12]. Previous research has confirmed the antidiabetic effects of *A. nilotica* [13] and the role of its polyphenolic contents in inducing hypoglycemia [11]. Legumes of this plant are known to cause hypoglycemia by stimulating insulin secretion in the islets of Langerhans through direct or indirect effects on β-cells [10]. Due to its natural antioxidant content, *A. nilotica* can be beneficial in the treatment of diseases such as cancer, DM, and inflammation which are caused by free radicals [11]. Tannin, or tannic acid, activates glucose transfer and prevents lipolysis [14]. Moreover, the hydroxyl groups in the phenolic compounds of *A. nilotica* turn this plant species into a potent free radical scavenger [15, 16].

To the best of our knowledge, no systematic review has ever evaluated the hypoglycemic effects of *A. nilotica* in type II DM. A systematic review provides an overview of primary studies, summarizes the research evidence and is best form of evidence. A systematic review protocol describes the rationale, hypothesis, and planned methods of the review too. It should be prepared before a review is started and used as a guide to carry out the review [17]. Therefore, the present paper describes a systematic review protocol for the assessment of the hypoglycemic effects of *A. nilotica*. Considering its high prevalence rate, the treatment of DM is a health priority according to the World Health Organization (WHO). We hence hope that the results of this study can confirm *A. nilotica* as a novel treatment for DM and a method for promoting the quality of life of patients with DM.

## Main text

### Methods/design

#### Registration and methodology

The study protocol was registered in the International Prospective Register of Systematic Reviews (PROSPERO) at the National Institute for Health Research (CRD42016053141). The guidelines of PRISMA-P (Preferred Reporting Items for Systematic Review and Meta-Analysis Protocols) were followed while reporting the study protocol.

### Inclusion criteria for studies

#### Type of study

All human or animal studies, including randomized and non-randomized placebo-controlled clinical trials, performed during 1999–2016 will be included. Research with cross-sectional and clustered designs, with or without blinding, will be evaluated. Case reports, as well as quasi-experimental and observational studies, will be excluded. No language limitations will be imposed and papers in languages other than English and Persian will be translated before use.

### Participants

#### The human studies will be eligible if their participants:

1. Aged 18 years and older at the time of type II DM diagnosis;
2. Underwent fasting blood sugar (FBS) or hemoglobin A1c (HbA1c) level measurements, glucose challenge test (GCT), glucose tolerance test (GTT), oral GTT (OGTT), or Glucose, 2 h Post Prandial (2HPP) to confirm hyperglycemia;
3. Used *A. nilotica* to induce hypoglycemia;
4. Used *A. nilotica* or placebo for at least 4 weeks; and
5. Completed the treatment course at a rate of more than 70%.

#### The animal studies will be eligible if their participants:

1. The induced diabetic—animal’s which recived *A. nilotica* (regardless to the type of diabetes induce).No limitation in sex or race of animals.
2. FBS, OGTT, GCT, and GTT were measured to assess blood glucose levels in animals.

#### Types of intervention

During the preliminary analysis, studies will be included if they involved:

1. The use of *A. nilotica* as the intervention;
2. The use of placebo in the control group; and
3. The use of placebo to alleviate symptoms [18]

#### Primary outcome

The rate of response to treatment (e.g. reductions in blood glucose levels) in the intervention and placebo groups will be regarded as the primary outcome. The outcomes will be measured through FBS, GCT, GTT, and OGTT in all of studies and in addition to these tests, will be measured 2HPP and HbA1c level in human study.

#### Secondary outcome

The side effects of *A. nilotica*, determining the most effective plant part, and the mean FBS or HbA1c, GCT, GTT, OGTT, or 2HPP levels will be considered as secondary outcomes.

#### Search strategies for selecting relevant studies

A search strategy will be adopted to find both published and unpublished articles. The Cochrane Central Register of Controlled Trials, MEDLINE, Google Scholar, EMBASE, ProQuest, Scopus, PsycINFO, and CINAHL databases will be searched using a number of key terms including *A. nilotica*, hypoglycemia, *A. arabica*, diabetes, and type II diabetes. Boolean operators “and” and “or” will be applied to make combinations of key terms. The search will involve three stages. During the first stage, a limited preliminary search using some of the mentioned key terms will be conducted in MEDLINE and CINAHL. Relevant articles will be selected based on their title, abstract, and keywords. In the second stage, all databases will be searched using all key terms. Finally, the references of all reports and articles will be searched as additional resources which were not listed in bibliographic databases. In order to find unpublished studies, government reports, protocols, gray literature, and student theses indexed in ProQuest will be searched.

#### Database of ongoing clinical trials

The following databases will be searched to find ongoing clinical trials:

- http://www.controlled-trials.com;
- http://www.clinicaltrials.gov; and
- http://www.who.int/trialsearch.

#### Searching other resources

Key journals in the field will be manually searched. As mentioned earlier, government reports, student theses, studies published by different research committees, and abstracts presented at various conferences and seminars will also be assessed.

### Data collection and analysis

#### Selection of relevant studies

The author (L.D) will initially evaluate the eligibility of the selected studies by reviewing their titles and abstracts. The two co-authors (F.A and N.R) will then assess the eligibility of the papers by independently evaluating their full texts. The authors will discuss their viewpoints to resolve any cases of disagreement. An external evaluator will be consulted if the discussions fail. Authors of papers whose abstracts are presented on posters will be contacted and asked to send the full text of their papers if possible.

#### Data extraction and management

Two co-authors (F.A and N.R) will individually assess full texts to extract the required data and enter them into a relevant form. The following pieces of information will be collected [19].

1. Research characteristics including the first author’s name, location and dates of publication and conduct of the study, research design, sample size, and duration of follow-up;
2. Patient characteristics including their age and gender, number of participants, the inclusion and exclusion criteria, keywords definitions, and measurement tools;
3. Intervention details including number of groups, blinding procedure, dose, duration, and type of intervention, determinants of treatment length, causes of treatment discontinuation, and sample loss); and
4. Outcome measures including details about the tools and methods used for the measurement of outcomes, side effects, and serious side effects.

The collected data will be reviewed by the third author and cases of disagreement will be resolved by consulting an external evaluator.

#### Quality assessment of studies

For quality assessment for human study, the Cochrane Risk of Bias Tool and for animal study the SYRCLE (Systematic Review Centre for Laboratory animal Experimentation) checklist will be use. Two external evaluators will independently. Again, an external evaluator will be consulted if any disagreements occur [18].

#### Data synthesis

Whenever possible, quantitative data will be pooled in statistical meta-analysis using STATA software. Two models of meta-analysis, i.e. the fixed-effect model and the random-effect model, will be used for outcomes. The fixed-effect model (which is based on the Mantel–Haenszel method) assumes that research samples are taken from populations with the same effect size. It thus weights studies according to the in-study variance. In contrast, the random-effect model assumes research samples are selected from populations with different effect sizes and weights studies based on both in-study and between-study variances (based on their level of heterogeneity). The latter model is more suitable in cases of higher heterogeneity. We will apply Chi square-based Q statistic to determine the between-study heterogeneity for each model. All results will be subject to double data entry. Effect sizes will be presented as odds ratio for categorical data and weighted mean differences for continuous data. Analysis will be performed on 95% confidence intervals of the calculated effect sizes. Chi square tests will be used to evaluate heterogeneity. Whenever statistical pooling is impossible, the findings will be merely provided in tables and figures. Meta-analyses will be performed on animal and human studies separately and the results of analyze will be compared in two groups. Subgroup meta-analyses based on outcome will also be conducted.

## Discussion

This study reviews the various aspects of hypoglycemic effects *A. nilotica* and its application in the treatments of type II diabetes. Systematic reviews will provide the highest level of evidence for informed decisions. A systematic review can provide convincing evidence relevant to many aspects of small set of studies. To the best of our knowledge, no systematic review has been conducted on this topic. Efforts will be made to publish the results in valuable peer-reviewed journals. Although, the lack of studies on the hypoglycemic effects of *A. nilotica* in type II diabetes has been given a study limitation.

We believe that our findings will provide details about difficulties researchers face during the design of protocols or implementation of scientific studies. The results will also help future studies by clarifying the characteristics of patients with type II DM who were recruited in previous research. Ultimately, the publication of our findings will facilitate the development of effective treatment strategies to promote the health of people with type II DM. One limitation of this study is the lack of studies about hypoglycemic effects of *A. nilotica* in type II diabetes.

### Limitations

In this study, the lack of studies on the hypoglycemic effects of *A. nilotica* in type II diabetes has been given a limitation. Another limitation is that the authors are only fluent in Persian and English. Therefore, a translator will be required when the papers are published in other languages.

## Abbreviations

DM: Diabetes Mellitus
WHO: World Health Organization
PROSPERO: Prospective Register of Systematic Reviews
FBS: Fasting Blood Sugar
HbA1c: Hemoglobin A1c
GCT: Glucose Challenge Test
GTT: Glucose Tolerance Test
OGTT: Oral GTT
2HPP: 2 h Post Prandial
